# Focus on a CKD-Associated Locus: *SHROOM3* and Other Suspects

**DOI:** 10.1016/j.ekir.2025.03.037

**Published:** 2025-03-28

**Authors:** Long Qian, Anand C. Reghuvaran, Madhav C. Menon

**Affiliations:** Department of Internal Medicine, Section of Nephrology, Yale School of Medicine, New Haven, Connecticut, USA

See Clinical Research on Page 1495

The genetic basis of chronic kidney disease (CKD) remains incompletely understood. Genome-wide association studies have repeatedly identified loci on chromosome 4q21.1 to be associated with lower estimated glomerular filtration rate (eGFR) and CKD,^1^^,^^2^ and most of these identified risk variants are located in the first intron of *SHROOM3*. With any noncoding variant identified by genome-wide association studies, the downstream mechanisms are difficult to pinpoint, and very likely involve regulatory effects on gene expression. Because 3 other genes, namely *CCDC158*, *STBD1*, and *FAM47E*, reside in this locus, a question raised is whether the identified variants in *SHROOM3* regulate *SHROOM3* itself or adjacent genes. Furthermore, whether the genome-wide association studies single-nucleotide polymorphisms (SNPs) are themselves tagging SNPs to other culprit SNPs in the region is unclear. Identifying the true causal gene and mechanism underlying the association of SNPs with CKD susceptibility could inform clinical strategies for screening, monitoring, risk stratification, and even therapeutics.

Overall, several lines of evidence support *SHROOM3* as the culprit gene in this locus. First, *SHROOM3* intronic SNPs are associated with CKD, acute kidney injury, and albuminuria.^3^ Next, *SHROOM3* expression in renal transplant biopsies correlated with the later development of graft fibrosis.^4^ In mechanistic studies, *SHROOM3* knockout mice had altered glomerulogenesis, whereas podocyte-specific *SHROOM3* knockdown in adult mice caused reversible albuminuria mediated by its interaction with FYN.^5^ In contrast, tubular knockdown of *SHROOM3* was protective in renal fibrosis, showing a dichotomy in its role in renal fibrosis versus podocyte function. A putative candidate culprit SNP in this region is intronic rs17319721, which was identified as a *TCF7L2*-dependent enhancer element for *SHROOM3* expression,^4^ or independently as a transcriptional start site for a shorter *SHROOM3* isoform associated with transcription of coding variants such as P1244L, potentially involved in CKD.^6^ These experiments have provided insight into downstream signaling pathways directly modulated by altered *SHROOM3* expression, such as Wnt/Ctnnb1, Tgfb1, and Hippo pathways.^3^

Potentially, antagonizing *SHROOM3* or downstream mechanisms in patients with these CKD-risk alleles could be a strategy to inhibit renal fibrosis in native and transplant CKD. However, current evidence may be insufficient for such an approach. For example, the risk allele rs17319721 (allele frequency: 0.39) confers a modest 10% to 15% increased risk for CKD.^1^^,^^2^ Not all carriers of this variant develop CKD, suggesting a heterogeneous effect. Incompletely understood gene-gene and/or gene-environment interactions likely facilitate the conversion of genotype to phenotype. A greater understanding of the genetic architecture of this region, and the phenotypic traits that modify disease risk in the presence of specific genotypes, would enhance the feasibility of potential precision medicine approaches.

In this issue of the journal, Ghasemi-Semeskandeh *et al.*^7^ aimed to delve deeper into variants at the chromosome 4q21.1 locus (encompassing all 4 genes) and CKD risk, by leveraging a large patient database to associate linked genomic variants with clinical traits. This study analyzed data from 12,834 participants in the Cooperative Health Research in South Tyrol study, a population-based cohort from northern Italy that includes extensive clinical, laboratory, and metabolic phenotyping, along with genomic data.^8^ Of these, 3840 underwent whole-exome sequencing, whereas the rest had targeted genotyping, with their whole-genome sequences imputed using the whole-exome sequencing data. The analysis focused on the 4q21.1 locus, identifying 71 single-nucleotide variants (synonymous, missense, and intronic), which were grouped into 11 haplotypes based on linkage disequilibrium.

Associations between haplotypes with participants’ clinical and laboratory traits were assessed, focusing on traits known to be associated with variants at this locus, such as kidney function. Compared with the reference haplotype, haplotypes 4, 6, 7, 8, and 9 correlated with higher eGFR or lower serum creatinine. Haplotypes 4 and 6 were associated with higher albuminuria. The paradoxical relationship of intronic *SHROOM3* SNPs and *SHROOM3* itself with lower GFR as well as lower albuminuria has been reported before,^5^ and reflects cell-specific pleiotropic effects of this gene, and is consistent with this study. To further explore potential mechanisms, the study examined associations between the variants and haplotypes and circulating protein expression levels in a subset of 4087 participants. The proteins encoded by genes within this locus (*SHROOM3*, *CCDC158*, *STBD1*, and *FAM47E*) were not directly measured. Interestingly, some identified haplotypes showed association with other proteins such as NAR3, CXCL11, and NAAA, which warrants further functional investigation. CXCL11, a chemokine, is also located on chromosome 4q21.1, and if these haplotypes prove to be true protein quantitative trait loci (pQTLs) for CXCL11 levels, this relationship may be a potential modifier of the effect of culprit SNPS on CKD. Lastly, the authors investigated genetic associations with blood metabolite levels in a subset of 7252 participants. Several haplotypes correlated with lower serum levels of specific metabolites, particularly carnitines. For example, haplotype 6 was associated with lower concentrations of dodecenoylcarnitine, hydroxyvalerylcarnitine, and tiglylcarnitine. To determine whether these metabolic associations were driven by the same pathways underlying the clinical traits, the authors conducted a mediation analysis, and found partial mediation for haplotype 6, where its effects on carnitine levels were attenuated after adjusting for serum creatinine or eGFR, suggesting shared biological pathways.

Altogether, this study provides some insights into how variants at this locus may relate to CKD. Notably, haplotype 6, which was associated with higher eGFR, contained rs10006043; this variant is in relatively low linkage disequilibrium with the CKD-associated A-allele at rs17319721 (R^2^ ∼ 0.5) even within European populations. Given that the reference haplotype was associated with lower GFR, the reference haplotype likely encompasses the previously reported CKD-risk variants, providing indirect, albeit weaker validation of previous genome-wide association studies. In addition, the association of haplotype 6 with lower carnitine levels may suggest a shared pathway underlying both carnitine levels and eGFR; and will inform future studies.

The authors’ approach of relating haplotypes to continuous clinical traits using multidimensional sources, including clinical data, proteomics, and metabolomics, is novel especially in the context of *SHROOM3*-region SNPs. The large sample size is also a strength. The study improves our understanding of the genetic architecture of the *SHROOM3* gene region in northern Italians. However, the identified haplotypes likely reflect geographical clustering, and their generalizability to a more diverse pan-European cohort requires further validation. It is also important to consider that genotypes of most of the cohort here were imputed rather than directly genotyped. Finally, it is critical to emphasize (as also acknowledged by the authors) that mechanisms of how any of these variants result in the clinical phenotypes and metabolic profiles remain ultimately unclear and requires mechanistic studies focused on each gene and each variant. Because the expression data reported in this study are from bulk experiments, cell type–specific modulation of gene expression by the SNPs cannot be extrapolated. For example, *SHROOM3* expression during CKD differs by cell type; with the loss of GFR, its expression increases in the nonglomerular compartment but falls in the glomerular compartment.^5^ Aligned with this, a recent functional genomics study^9^ identified proximal tubular cells as the likely site where risk-associated variants regulate *SHROOM3*. These studies highlight the need for cell type-specific investigations.

Overall, by integrating genomic, clinical, proteomic, and metabolic data, this study provides indirect support for the link between variants at the Chr 4q21.1 locus and *SHROOM3* with renal phenotypes and suggests the need to probe future datasets for genetic variants as well as environmental factors, to fully unravel the association of *SHROOM3* risk genotypes with CKD.

## Disclosure

MCM is an inventor on provisional patent “Methods and Compositions for Identifying, Characterizing, and Treating Kidney Diseases/Disorders: Nephro-Dx test. [U.S.S.N. 63/757,108]” which includes *SHROOM3*. All the other authors declared no competing interests.
